# Sexual size and shape dimorphism in *Brachydesmus troglobius* Daday, 1889 (Diplopoda, Polydesmida)

**DOI:** 10.3897/zookeys.930.48285

**Published:** 2020-04-28

**Authors:** Vukica Vujić, Luka Lučić, Sofija Pavković-Lučić, Bojan Ilić, Zvezdana Jovanović, Slobodan Makarov, Boris Dudić

**Affiliations:** 1 Faculty of Biology, Institute of Zoology, University of Belgrade, Belgrade, Serbia University of Belgrade Belgrade Serbia

**Keywords:** flat-backed millipedes, geometric morphometrics, intersexual morphological differences, polydesmidan millipedes, sexual shape dimorphism

## Abstract

Until now, morphological trait variation has been investigated in several millipede species using geometric morphometrics. The present study is the first attempt to explore sexual shape and size dimorphism (SShD and SSD) of morphological structures in Polydesmida. We here analyse antennal, head, and leg SShD and SSD in *Brachydesmus troglobius* Daday, 1889. Our results show that SSD exists in all of the analysed structures, while SShD is present only in the legs. In comparison with females, males possess longer and wider legs, as well as longer antennae and a shorter head. Contrary to previous findings in some Julida, in *B. troglobius*SSD of the antennae and legs varies more than SShD in these morphological structures.

## Introduction

Sexual dimorphism (SD) is frequently studied in many biological fields and refers to any morphological, behavioural, physiological, and lifespan differences between the sexes (Fairbairn et al. 2007; Austad and Fischer 2016; Janicke et al. 2016). Besides sexual selection, the origin and maintenance of various forms of SD can be related to ecological factors (i.e., sex-specific interactions with the natural environment) or different behavioural traits (i.e., parental care, locomotor activity before mating, etc.) (Slatkin 1984). Intersexual morphological differences have been widely investigated in many arthropods (Walker and Rypstra 2002; Cooper 2014, 2016, 2017, 2018a, b; Virginio et al. 2015; Bidau et al. 2016; Medina et al. 2016; Ilić et al. 2017, 2019; Rohner et al. 2018). Secondary sexual traits were mostly investigated in these studies (Markow 1994; Watson and Simmons 2010).

Both sexual size and shape dimorphism (SSD and SShD, respectively) of morphological traits represent components of SD since both of them may be under different evolutionary pressures in females and males. To describe SD precisely, it is necessary to analyse both of the mentioned components (Berns 2013). Despite this fact, SShD was rarely investigated in comparison with SSD in numerous zoological studies (Cheng and Kuntner 2015). Like intersexual differences in size, shape differences can result from sex-specific behavioural peculiarities and ecological differences arising from specific ecological demands of the sexes (Butler and Losos 2002).

Millipedes represent one of the first arthropods colonizing terrestrial habitats. There is a need for better understanding of the morphological intersexual architecture of these ancient animals. Intersexual differences in the following traits have been investigated in several groups of millipedes: number of leg pairs and body segments (Verhoeff 1928; Mauriès 1987; Minelli and Michalik 2015); morphology and setation of the metaterga (Minelli and Michalik 2015; VandenSpiegel and Golovatch 2015); body size and body mass (Enghoff 1982; Adolph and Geber 1995; Rowe 2010; Cooper 2014, 2018a,b; Ilić et al. 2017); antennal length (Enghoff 1982; Ilić et al. 2017); leg length (Enghoff 1982; Rowe 2010; Ilić et al. 2017); head and trunk size (Ilić et al. 2017); morphology of anterior legs and mandibles (Minelli and Michalik 2015) and the gnathochilarium (Ilić et al. 2017); and that of the coxal glands (Hopkin and Read 1992). Further, SSD of body length, body mass, trunk height, trunk width, and antennal and leg centroid size (CS) was investigated in three diplopod species: *Pachyiulus hungaricus* (Karsch, 1881), *Megaphyllum unilineatum* (C.L. Koch, 1838), and *M. bosniense* (Verhoeff, 1897) (Ilić et al. 2019). In previous studies, shape differences of morphological traits were analysed using different methods. Specifically, in some julid species, Enghoff (1982) described body shape as the ratio between certain linear measurements, while Ilić et al. (2019) used the geometric morphometric technique (GM) to explore SShD. However, GM has never before been used to describe SShD of morphological traits in other diplopod groups, including Polydesmida.

In the present work, *Brachydesmus troglobius* Daday, 1889 was selected as a model-system to analyse SSD and SShD of three morphological structures, namely antennae, heads, and legs. Bearing in mind that it was previously shown that individuals of the sampled population of *B. troglobius* were in different phases of the life cycle in the Lazareva Pećina Cave (Ćurčić and Makarov 1998), we here analysed whether such life history differences influence the SSD and SShD of some morphological traits in this millipede species. The Lazareva Pećina Cave represents a complex underground system consisting of three levels (the two upper levels are fossilized, while the lowest one still functions as a permanent stream). The main corridors of this cave originated at the end of the Pliocene (Petrović 1958), but formation of the underground karst relief in this region had started already in the Lower Miocene. It is possible that colonization of the population of *B. troglobius* in the Lazareva Pećina Cave is not a recent event.

To our knowledge, this study represents the first attempt to analyse SSD and SShD of the head in Polydesmida. Bearing in mind the role that these body parts have during mating behaviour in Polydesmida (Snider 1981; Rowe 2010), we hypothesized that SSD and SShD exist in all of the aforementioned traits.

## Materials and methods

**Figure 1.**
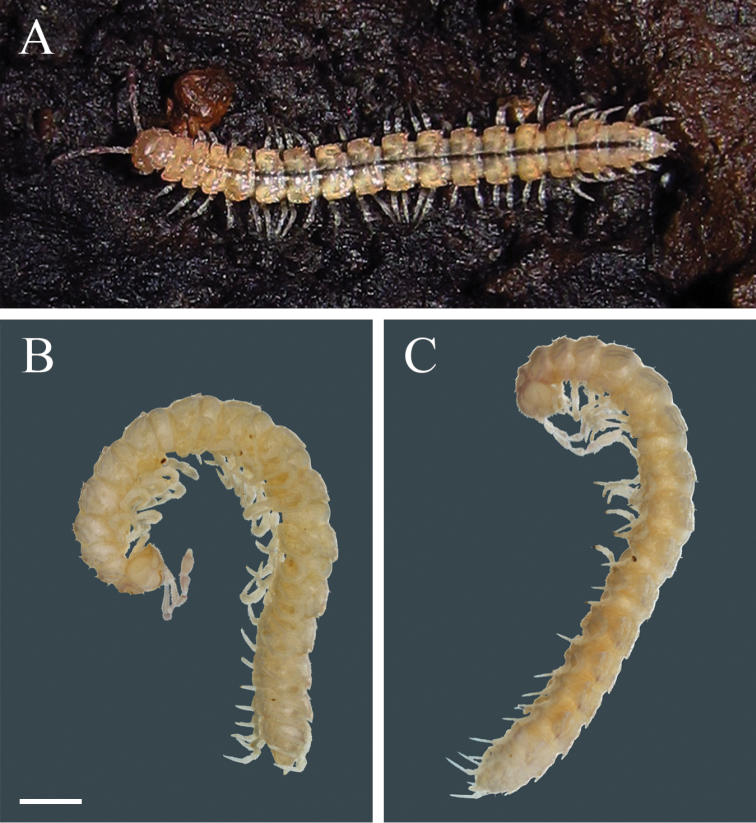
*Brachydesmus troglobius* Daday, 1889 **A** male photographed in Lazareva Pećina Cave **B** male **C** female. Photo credit: D. Antić (**A**), V. Vujić and B. Ilić (**B, C**). Scale bar: 1 mm (**B, C**).

**Figure 2.**
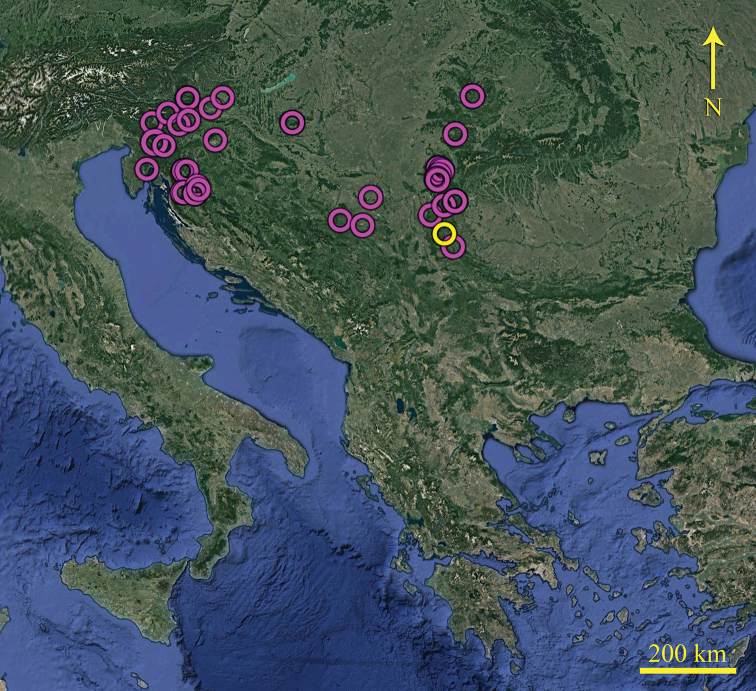
Distribution of *B. troglobius* (yellow circle- Lazareva Pećina Cave, and purple circles- literature records of *B. troglobius*).

*Brachydesmus troglobius* (Fig. 1A–C) is frequently found in caves, but also in epigean habitats in some European countries (Fig. 2) (Strasser 1971; Mršić 1985, 1988, 1994; Ćurčić and Makarov 1998; Tabacaru et al. 2002–2003; Makarov et al. 2004; Korsós et al. 2006; Antić et al. 2013; Angyal and Korsós 2013; Angyal et al. 2017). The analysed species belongs to the genus *Brachydesmus* Heller, 1858, which includes numerous species and subspecies (in many cases with dubious validity) with great diversity on the Balkan Peninsula (Makarov et al. 2004; Antić et al. 2013). In the present study, samples were collected at the main corridor (300 m from the entrance) of Lazareva Pećina Cave (eastern Serbia). The samples were collected during the 1997/1998 season. All specimens used in this study were preserved in 70% ethanol immediately after collecting and deposited in collections of the Institute of Zoology, University of Belgrade – Faculty of Biology. Three morphological structures (antennae, heads, and legs) were dissected and further used for analysing SSD and SShD.

**Figure 3.**
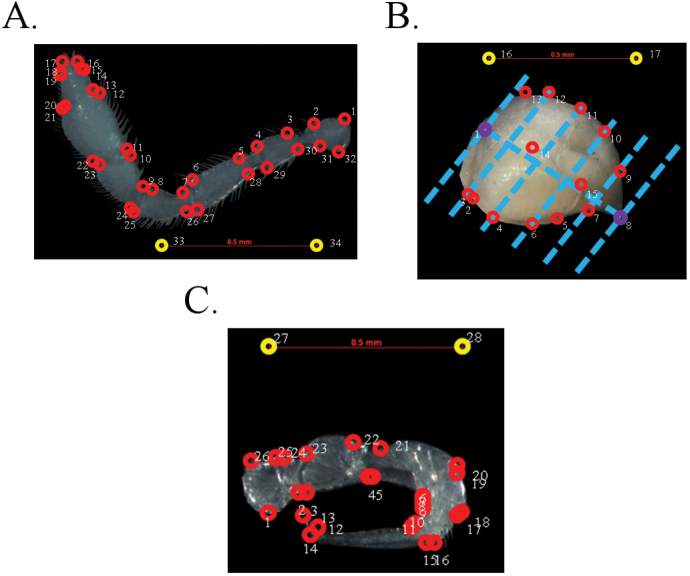
Position of landmarks and semi-landmarks (3, 4, 6, 7, 9–12 on the picture of head) on the analysed morphological structures **A** antenna **B** head **C** leg.

Size and shape of the left and right antennae (average value of both antennae, in 28 females and 21 males), heads (in 27 females and 22 males), and both legs from the anterior leg-pair of the 10^th^ body ring (average value of both legs in 28 females and 21 males) were analysed. First of all, each morphological structure was dissected using a Carl Zeiss Stemi-2000 binocular stereomicroscope. Photos of all morphological structures were taken with a Carl Zeiss Axiocam MRc camera. The Make Fan program (available at http://www3.canisius.edu/~sheets/IMP%208.htm) was used to create fans on each picture of the heads. In the TpsDig program (Rohlf 2008, available at http://life.bio.sunysb.edu/morph/soft-dataacq.html), 32 landmarks were positioned on pictures of antennae, 10 semi-landmarks and 5 landmarks were positioned on pictures of heads (lateral view), and 26 landmarks were positioned on each picture of legs (Fig. 3A–C). Centroid size (CS) for each morphological structure was calculated in the CoordGen6 program (Sheets 2003, available at http://www3.canisius.edu/~sheets/IMP%208.htm). Sexual shape differences were analysed using Canonical Variate Analysis (CVA), performed in the MorphoJ program (Klingenberg 2011, available at http://www.flywings.org.uk/morphoj_page.htm). Statistica 7 (StatSoft, Tulsa, OK, USA) was used to test intersexual differences in the CS of antennae, heads, and legs. The R program (R Core Team 2013) was used to visualize differences in CS values of the aforementioned traits. The distribution map was created using Google Earth Pro (version 7.3.2.5776).

## Results

**Figure 4.**
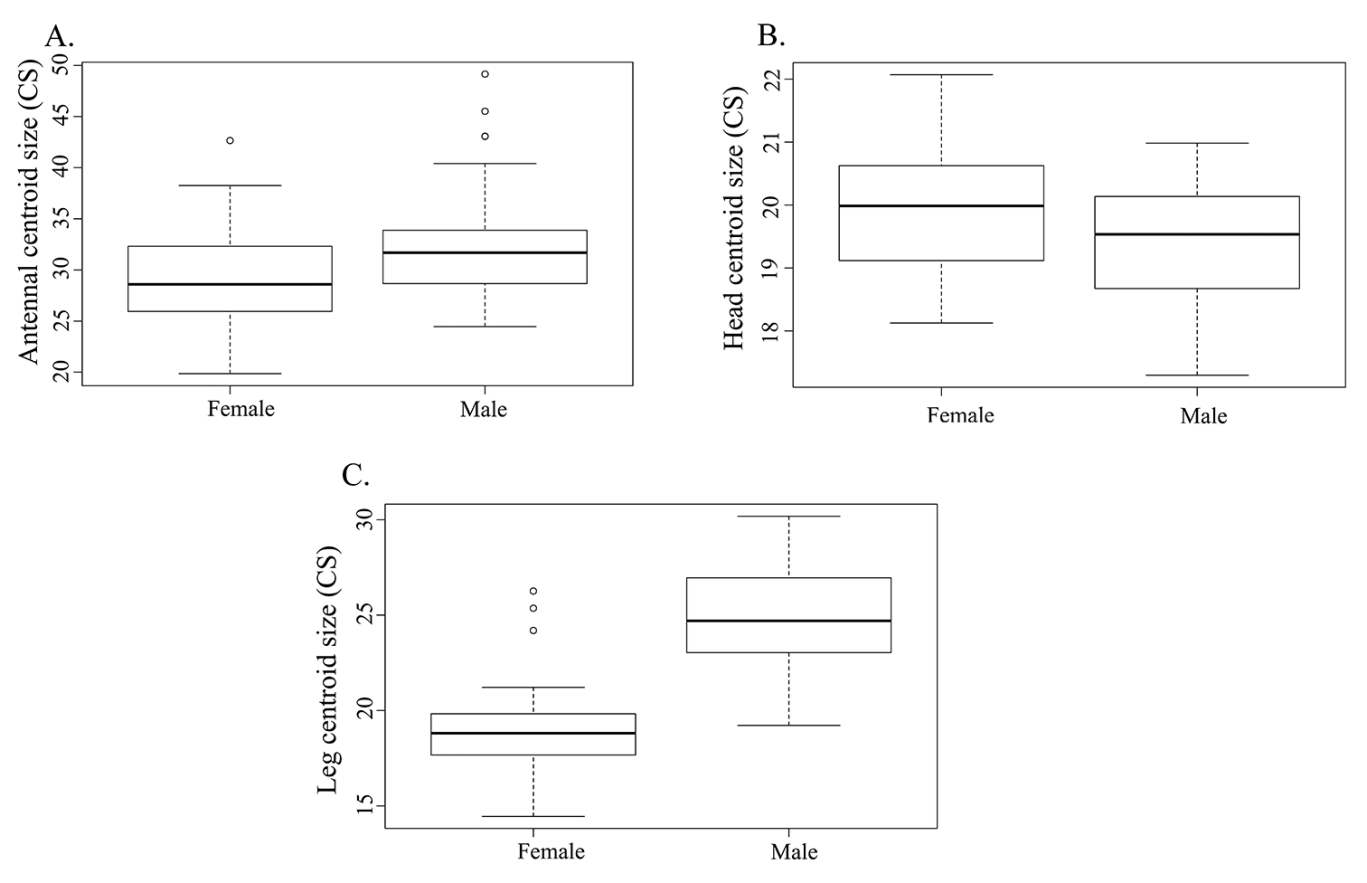
Intersexual differences in CS of: **A** antennae **B** heads **C** legs.

**Figure 5.**
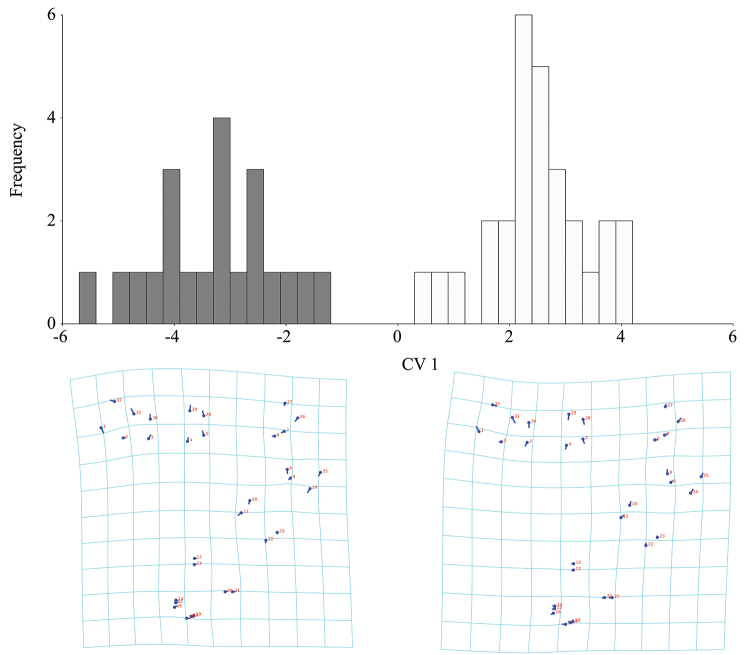
Intersexual differences of antennal shape in *B. troglobius* illustrated using Canonical Variate Analysis (CVA). Position and size of the vectors’ influence on a thin-plate spline deformation grid and illustration of the pattern of intersexual differences of antennal shape (white bars indicate females; grey bars indicate males).

**Figure 6.**
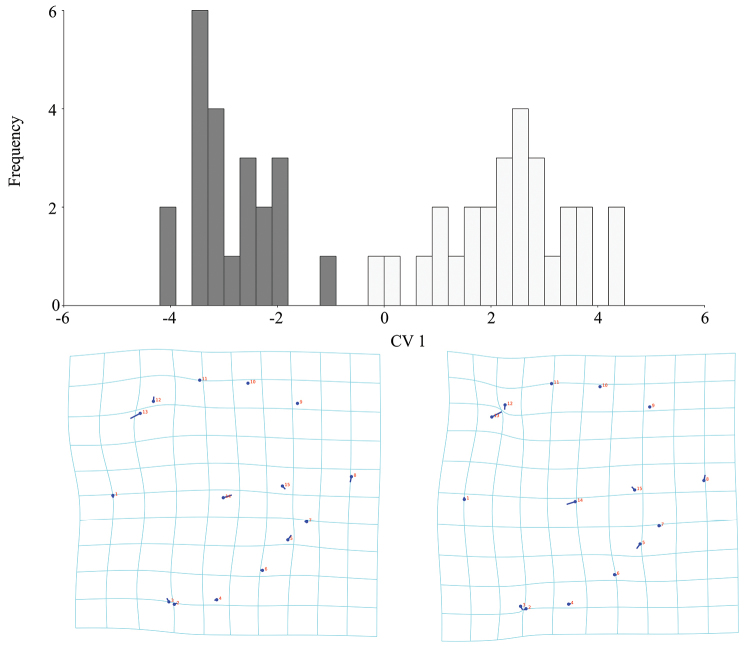
Intersexual differences of head shape in *B. troglobius* illustrated using Canonical Variate Analysis (CVA). Position and size of the vectors’ influence on a thin-plate spline deformation grid and illustration of the pattern of intersexual differences of head shape (white bars indicate females; grey bars indicate males).

**Figure 7.**
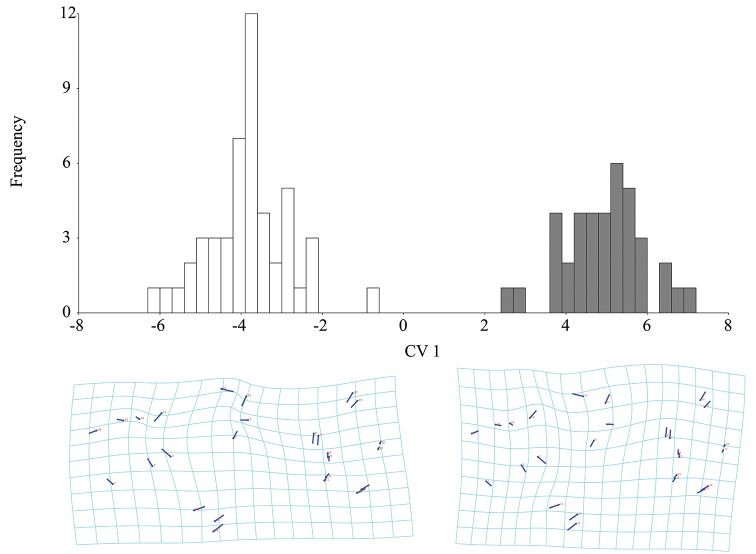
Intersexual differences of leg shape in *B. troglobius* illustrated using Canonical Variate Analysis (CVA). Position and size of the vectors’ influence on a thin-plate spline deformation grid and illustration of the pattern of intersexual differences of leg shape (white bars indicate females; grey bars indicate males).

Intersexual differences of CS were present in all analysed structures (antennae: *p* = 0.0081; heads: *p* = 0.0481; legs: *p* < 0.0001) (Fig. 4A–C). Sexual shape dimorphism was present only in legs (antennae: *p* = 0.6319; heads: *p* = 0.0882; legs: *p* = 0.0008) (Figs 5–7). Males possess longer and wider legs in comparison with females (Figs 4C, 7), as well as longer antennae (Fig. 4A), while the opposite pattern was observed in analysis of intersexual differences in head CS (Fig. 4B).

## Discussion

In polydesmidan millipedes, SShD has never been studied using both traditional and GM techniques. However, SSD in polydesmidan species has been investigated using linear body measurements (length and width), body mass, and leg length (Adolph and Geber 1995; Rowe 2010). Sexual size dimorphism in the aforementioned morphological traits has been examined in some millipede species. Intersexual differences of body dimensions and mass were investigated in two polydesmidan species, viz., *Nyssodesmus python* (Peters, 1864) (Adolph and Geber 1995) and *Cladethosoma clarum* (Chamberlin, 1920) (Rowe 2010); the callipodidan species *Apfelbeckia insculpta* (C.L. Koch, 1867) (Ilić et al. 2017); the julidan species *Cylindroiulus* sp. (Enghoff 1982) and *Pachyiulus hungaricus*, *Megaphyllum bosniense*, and *M. unilineatum* (Ilić et al. 2019); and the spirobolidan species *Chersastus* sp. (Cooper 2014) and *Centrobolus inscriptus* (Attems, 1928), *Ce. fulgidus* (Lawrence, 1967), *Ce. ruber* (Attems, 1928), and *Ce. diagrammus* (Pocock, 1893) (Cooper 2018c). Also, SD of trunk dimensions was studied in *A. insculpta* (Ilić et al. 2017); and in *P. hungaricus*, *M. bosniense*, and *M. unilineatum* (Ilić et al. 2019). Sexual dimorphism of leg length, which is described as total length and/or length of individual podomeres, was investigated in *Cl. clarum* (Rowe 2010); in *A. insculpta* (Ilić et al. 2017); and in *P. hungaricus*, *M. bosniense*, and *M. unilineatum* (Ilić et al. 2019), as well as in several species of the genus *Cylindroiulus* (Enghoff 1982). Likewise, SD of antennal length has been analysed in all of the aforementioned species with the exception of *Cl. clarum*. Also, SD of the head and gnathochilarium was studied using traditional morphometric technique in the case of *A. insculpta* (Ilić et al. 2017). Additionally, in some of these studies, the shape of several morphological structures was described using different techniques. Thus, shape was described using only linear measurements in callipodidan species (Ilić et al. 2017), while shape variation was described using both ratios of linear measurements and the GM approach in some julidan species (Enghoff 1982; Ilić et al. 2019). Results obtained using the GM technique in the present study revealed that in *B. troglobius*SSD is present in all of the examined structures (antennae, heads, and legs), while SShD is present only in the legs. Our results indicated that females have shorter and narrower legs as well as shorter antennae than males. Rowe (2010) provided an explanation for the presence of longer legs in males based on positive correlations between leg length and motion speed, i.e., between leg length and the mate encounter rate. Thus, males with longer legs can find a suitable partner for mating more quickly. Also, longer legs in Polydesmida species could be important for mating success, since the male during mating firmly grasps the female with his legs (e.g., Harz 1962; Snider 1981; Tanabe and Sota 2008).

In addition, we found that females possess higher values of head CS in comparison with males, which is in agreement with the previously reported situation in the case of *A. insculpta* (Ilić et al. 2017). This result can be attributed to the presence of fecundity selection, implying that females spend more time feeding and/or nest building in comparison with males, which spend more time finding suitable mating partner. The females of *B. troglobius* are the larger sex and we presume that they invest more in offspring. Besides SSD, in the present study we also analysed SShD of the antennae, heads, and legs. Although intersexual differences of antennal and leg shape were previously studied using GM (Ilić et al. 2019), this is the first GM-based report on intersexual differences of head shape in millipedes.

The GM approach has been widely used to describe intersexual differences of morphological traits in arthropods (Benítez 2013; Fernández-Montraveta and Marugán-Lobón 2017; Gushki et al. 2018; Vesović et al. 2019). In millipedes, intersexual morphological differences were previously described by Ilić et al. (2019) using the GM technique. Our results indicated leg SShD in *B. troglobius*. This finding is in line with previously reported results indicating that leg SShD exists in some other julidan species, ones such as *P. hungaricus*, *M. unilineatum*, and *M. bosniense* (Ilić et al. 2019). In the case of antennal SShD, the results of our study are not concordant with previous findings in millipedes. In the present study, the presence of antennal SShD was not obtained in *B. troglobius*, whereas this pattern of SD was previously detected in two julidan species, *P. hungaricus* and *M. bosniense* (Ilić et al. 2019). As for antennal length SD, *B. troglobius* males possess longer antennae in comparison with females, whereas *P. hungaricus* and *M. unilineatum* males possess shorter antennae than females (Ilić et al. 2019). *Brachydesmus troglobius* males possess longer legs in comparison with females, whereas leg SD was not detected in three julidan species, *P. hungaricus*, *M. unilineatum*, and *M. bosniense* (Ilić et al. 2019). In our opinion, this discordance in leg length between our findings and previously reported results could be associated with the different life histories of julidan (mostly iteroparous) and polydesmidan (all semelparous) species (Blower 1985; Bhakat et al. 1989; David 1992 and references therein; Hopkin and Read 1992; Minelli 2015). As semelparous species seek to maximize fitness by investing all energy and gametes in a single breeding season (Narum et al. 2008), it is reasonable to expect a tighter relationship between mating success and morphological traits associated with it. Furthermore, longer legs in polydesmidan males could be linked with the presence of scramble competition polygyny in millipedes (Telford and Dangerfield 1993; Rowe 2010; Holwell et al. 2016). One of the male behavioural types included in this system is maximization of fitness through investment in mate acquisition (Herberstein et al. 2017). This explanation is also supported by the fact that there is a positive correlation between speed and leg length in millipedes (Manton 1973). Apart from analysis of SD using GM, there are several studies of SD based on analyses of linear measurements (Rowe 2010; Cooper 2016; Ilić et al. 2017). Our results are in agreement with previously reported findings in *Cl. clarum*, in which males possess wider and longer legs in comparison with females (Rowe 2010), and with results reported for the callipodidan species *A. insculpta* indicating that females possess shorter legs than males (Ilić et al. 2017).

With respect to the head, no SShD was observed in *B. troglobius*. Our results also showed that females of *B. troglobius* have a longer head than males, which is in agreement with the previously reported situation in the case of *A. insculpta* (Ilić et al. 2017). For antennal SSD, Ilić et al. (2017) noted that males of *A. insculpta* possess longer antennae than females, the same as the pattern detected in *B. troglobius*.

## Conclusion

No antennal SShD or head SShD was observed in the present study, although antennal and head SShD was present in some previously studied julidans, as well as head SShD in some callipodidans. However, leg SShD was detected in *B. troglobius*, in some julidan species, and one callipodidan species. The same patterns of intersexual differences of antennal and head length were detected in both *B. troglobius* and the callipodidan species *A. insculpta*.
